# *ZNF577* Methylation Levels in Leukocytes From Women With Breast Cancer Is Modulated by Adiposity, Menopausal State, and the Mediterranean Diet

**DOI:** 10.3389/fendo.2020.00245

**Published:** 2020-04-23

**Authors:** Paula M. Lorenzo, Andrea G. Izquierdo, Angel Diaz-Lagares, Marcos C. Carreira, Manuel Macias-Gonzalez, Juan Sandoval, Juan Cueva, Rafael Lopez-Lopez, Felipe F. Casanueva, Ana B. Crujeiras

**Affiliations:** ^1^Laboratory of Epigenomics in Endocrinology and Nutrition (EpiEndoNut), Instituto de Investigacion Sanitaria de Santiago de Compostela (IDIS), Complejo Hospitalario Universitario de Santiago de Compostela (CHUS/SERGAS), Santiago de Compostela, Spain; ^2^CIBER de Fisiopatologia de la Obesidad y Nutricion (CIBEOBN), Instituto de Salud Carlos III, Madrid, Spain; ^3^Cancer Epigenetics, Translational Medical Oncology (Oncomet), Health Research Institute of Santiago (IDIS), University Clinical Hospital of Santiago (CHUS/SERGAS), Santiago de Compostela, Spain; ^4^CIBER de Oncologia (CIBERONC), Instituto de Salud Carlos III, Madrid, Spain; ^5^Laboratory of Molecular and Cellular Endocrinology, Instituto de Investigacion Sanitaria de Santiago de Compostela (IDIS), Complejo Hospitalario Universitario de Santiago de Compostela (CHUS/SERGAS) and Santiago de Compostela University (USC), Santiago de Compostela, Spain; ^6^Department of Endocrinology and Nutrition, Virgen de la Victoria University Hospital, University of Malaga (IBIMA) and CIBEROBN, Málaga, Spain; ^7^Biomarkers and Precision Medicine Unit and Epigenomics Core Facility, Health Research Institute La Fe, Valencia, Spain; ^8^Translational Medical Oncology (Oncomet), Health Research Institute of Santiago (IDIS), University Clinical Hospital of Santiago (CHUS/SERGAS), Santiago de Compostela, Spain

**Keywords:** epigenetics, obesity, cancer, nutrition, blood cells, biomarkers

## Abstract

The methylation levels of *ZNF577* in breast tumors has been previously identified as a possible epigenetic mark of breast cancer associated with obesity. The aim of the current study was to investigate differences in methylation levels of *ZNF577* depending on obesity, menopausal state and dietary pattern in blood leukocytes, a non-invasive sample. The methylation levels of *ZNF577* of two CpG sites (CpGs) located in promoter and island previously identified as differentially methylated according to adiposity and menopausal state by 450 k array (cg10635122, cg03562414) were evaluated by pyrosequencing in DNA from the blood leukocytes of breast cancer patients [*n* = 90; *n* = 64 (71.1%) overweight/obesity and *n* = 26 (28.9%) normal-weight] and paired tumor tissue biopsies (*n* = 8 breast cancer patients with obesity; *n* = 3/5 premenopausal/postmenopausal women). Differences in methylation levels were evaluated at each CpGs individually and at the mean of the two evaluated CpGs. Adherence to the Mediterranean diet was evaluated using the MEDAS-validated questionnaire, and the consumption of food groups of interest was also evaluated using the recommended intakes of the *Sociedad Española de Nutricion Comunitaria*. The methylation levels of *ZNF577* were correlated between paired leukocytes and breast tumor biopsies (*r* = 0.62; *p* = 0.001). Moreover, higher methylation was found in leukocytes from patients with obesity (*p* = 0.002) and postmenopausal patients (*p* = 0.022) than patients with normal-weight or premenopausal, respectively. After adjusting for the body mass index and age, higher levels of *ZNF577* methylation were also found in women with greater adherence to the Mediterranean diet (*p* = 0.017) or specific foods. Relevantly, the methylation levels of *ZNF577* showed a good ability for fish consumption detection [area under the ROC curve (AUC) = 0.72; *p* = 0.016]. In conclusion, the association between methylation of *ZNF577* and adiposity, menopausal state, and adherence to the Mediterranean diet can be detected in the blood leukocytes. The results guarantee the need of performing further studies in longer longitudinal cohorts in order to elucidate the role of *ZNF577* methylation in the association between breast cancer, adiposity and dietary patterns.

## Introduction

Breast cancer is the leading form of cancer diagnosed in women (1, 2). Excess adiposity and dietary habits occupy a prominent position among the most relevant risk factors of breast cancer (3–5). Increasing scientific evidence demonstrates that environmental factors modulate the expression of genes by regulating epigenetic mechanisms (6, 7). Therefore, the effect of excess body weight and dietary factors on the promotion of breast cancer may be mediated by epigenetic regulation (8).

Our previous studies have recently evaluated the associations between the body mass and DNA methylation in obesity-related diseases (9–12). In the context of breast cancer, an epigenetic signature of obesity-related breast cancer has been identified in breast tumor biopsies, being *ZNF577* the most represented gene (10).

The functional role of *ZNF577* is unknown; however, some members of the zinc finger proteins (ZNFs) family, which regulate gene transcription, have been found to be often hypermethylated and silenced in different types of tumors, suggesting that it may represent a commonly disrupted epigenetic pathway in cancer progression (13). In a metabolic and nutritional context, family members of the ZNFs have been associated with adipogenesis (14–16), type 2 diabetes, insulin resistance (17, 18), the regulation of hepatic lipogenesis (19, 20), and in the control of muscle function (21). Moreover, the expression of some ZNFs is regulated by nutritional status such as fasting (22) or under a high-fat diet (23, 24). Recently it was observed that the consumption of fruit and vegetables concentrates modulate the expression of a ZNFs gene in overweight/obese subjects (25). However, to the best of our knowledge, few studies have evaluated the effect of food consumption on the regulation of the expression of genes encoding ZNFs in humans.

Elucidating potential effect of adiposity and dietary patterns on the epigenetic regulation of breast cancer may lead to a better understanding of this disease. Epigenetic mechanisms are involved in the pathogenesis of breast cancer and considering that obesity is a risk factor of breast cancer, changes of diet or body weight might reduce the risk of disease remodelating epigenetic marks. This is especially important as the global prevalence of obesity continues to rise (26, 27). In the area of nutrigenomics/nutriepigenomics, recently published studies have provided evidence of the suitability of studying DNA methylation in the blood leukocytes (11, 28–37).

In this study we evaluate the methylation levels of *ZNF577* because it was among the genes of the episignature of obesity-related breast cancer previously identified (10) and it could be a potential player in the link between obesity and breast cancer. Therefore, this study was first aimed to evaluate the capacity of circulating leukocytes to reflect the DNA methylation pattern of *ZNF577* in the breast tumor tissue. Furthermore, this study also aimed to assess the relationship between the adherence to the Mediterranean diet and effects of its specific constituents on the methylation and expression pattern of *ZNF577*.

## Subjects and Methods

### Study Participants

A total of 101 women newly diagnosed with histologically confirmed invasive breast cancer during 2010–2011 were included from the *Complejo Hospitalario Universitario de Santiago de Compostela* (CHUS). Patients were excluded if they had other disease different of breast cancer such as cardiovascular, renal or infectious disease. Among these patients, samples, complete clinical data and information on dietary habits were obtained for 90 women, and they were included in this study. Leukocytes were obtained from blood samples in the 90 women. Moreover, paired samples from breast tumor tissue biopsies were obtained in 8 breast cancer women with obesity (*n* = 3 premenopausal women and *n* = 5 postmenopausal women). Breast cancer human paraffin embedded (FFPE) tissue blocks were obtained from the Biobank of the *Complejo Hospitalario Universitario de Santiago de Compostela* (CHUS) (PT13/0010/0068), integrated in the Spanish National Biobanks Network and they were processed following standard operating procedures with the appropriate approval of the Ethical and Scientific Committees (ref 2009/076) (10).

Pre-diagnosis body weight, height, age, and menopausal status were retrieved from medical records for all participants in the study. Additionally, a questionnaire on the dietary habits was also obtained along with the blood samples in fasting. Body mass index (BMI) was calculated as weight in kg divided by the squared height in meters and was further categorized using the World Health Organization (WHO) criteria: normal/under-weight, BMI < 25 kg/m^2^; overweight, 25 ≤ BMI < 30 kg/m^2^; and obese, BMI ≥ 30 kg/m^2^ (38). Then, the overweight and obese patients were classified together as obese (BMI > 25 kg/m^2^), and the others as non-obese (BMI ≤ 25 kg/m^2^) to evaluate the effect of excess body weight on the methylation patterns.

All participants provided informed consent, and the informed consent and the study protocols were approved by the Institutional Review Boards of the participating institution (Comité Ético de Investigación Clínica, CEIC, de Galicia; Ref:2009/076).

### Dietary Assessment

The dietary intake of patients was evaluated using a validated self-referenced Food Frequency Questionnaire (FFQ). The questionnaire included 49 food and beverage items, as well as a small questionnaire about the type of oil used for cooking and dressing. Subjects were asked to specify their frequency of consumption for each food item on a daily, weekly, or monthly basis.

Intakes were then converted to daily frequencies and a manual for household measures was used to convert intake frequencies to grams of food intake/day (39). Considering the recommendations of food intakes of the *Sociedad Española de Nutricion Comunitaria* (SENC) (40), two groups were established for each food item (lower consumption than recommended & higher consumption than recommended). Also, with the data obtained in the FFQ, adherence to the Mediterranean diet was assessed based on 12 questions from the MEDAS-validated questionnaire (41). Under this condition two groups were established. First, patients who secured higher than 7 points from the 12 questions of the MEDAS questionnaire were categorized as the group with high adherence to the Mediterranean diet. Second, patients who secured 7 points or less were included in the group with low adherence to the Mediterranean diet.

### DNA Preparation and Bisulfite Conversion

Genomic DNA was isolated from fresh-frozen (FF) leukocytes (*n* = 90) and paired breast cancer human paraffin embedded (FFPE) tissue blocks (*n* = 8).

Genomic DNA was isolated from FF leukocytes by using the MasturePure^TM^ DNA purification kit (Epicentre Biotechnologies, Madison, WI, USA), while paraffin samples (FFPE) (10 sections of 14 μm) were processed using the E.Z.N.A. FFPE DNA kit (Omega Bio-Tek), with a xylene wash to remove the paraffin. For each sample of tumor tissue, subsequent sections were stained with hematoxylin and eosin for histologic confirmation of the presence (>50%) of tumor cells (10). The obtained DNA was treated with RNase A for 1 h at 45°C (Qiagen, Hilden, Germany). All DNA samples were quantified using the fluorometric method (Quan-iT PicoGreen DsDNA Assay, Life Technologies) and were assessed for purity using a NanoDrop 2000c (Thermo Fisher Scientific) with 260/280 and 260/230 ratio measurements. High-quality DNA samples (500 ng of FF and 300 ng of FFPE) obtained were selected for bisulfite conversion using the EZ-96 DNA Methylation kit (Zymo Research Corp.) following the manufacturer's recommendations.

### Pyrosequencing Analysis

**Figure 1:**
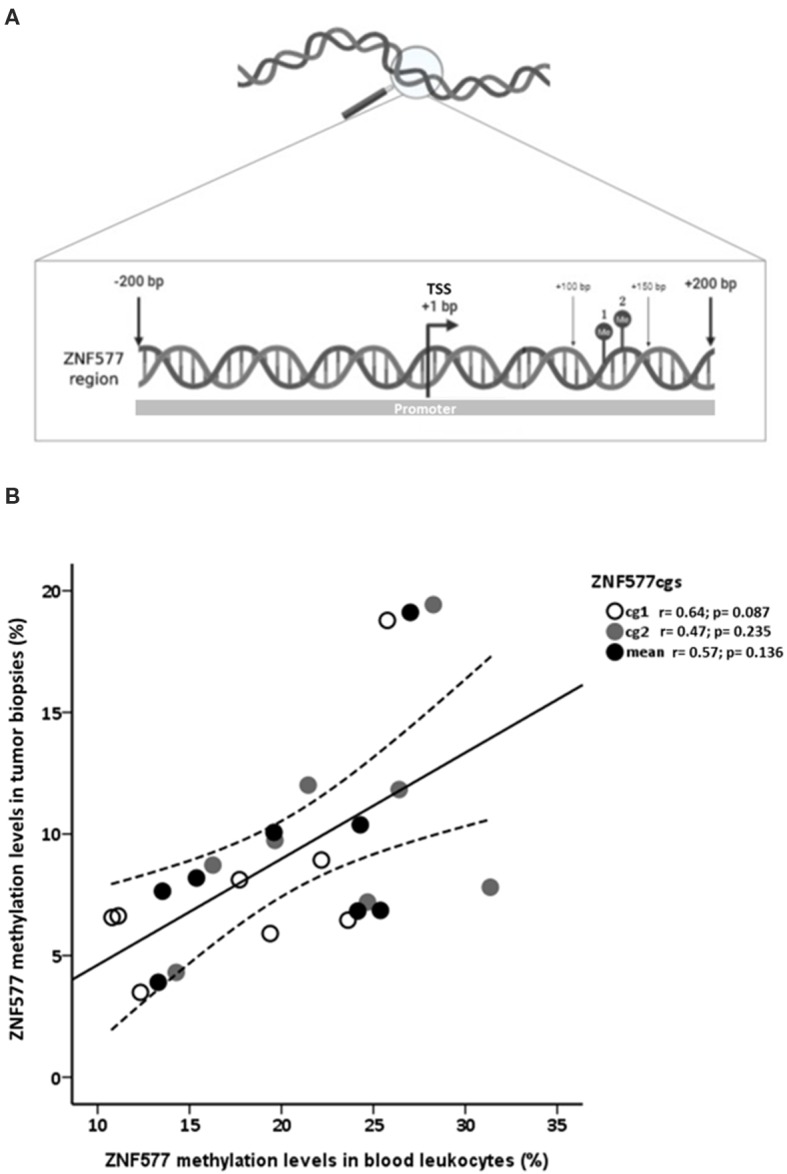
Analysis of methylation levels of *ZNF577*. **(A)** Map of a DNA fragment from the promoter of *ZNF577* gene with 200 nucleotides upstream (–) and 200 nucleotides downstream (+) of transcription start site (TSS) containing the examined CpG sites. Points 1 and 2 represent CpG sites located at the mapinfo 52391078 and 52391090, respectively, according to GRCh37/hg19 from UCSC Genome Browser and correspond to the target IDs cg03562414 and cg10635122 of the Infinium Human Methylation 450 BeadChip array. These CpG sites were previously identified as differentially methylated in breast tumor tissues depending on menopausal and adiposity state (10). The promoter region was defined as the sequence from 1,500 bps upstream of TSS to 1st exon (42, 43) **(B)** Scatterplot representing the correlation of *ZNF577* methylation levels of CpG1, CpG2 and mean in leukocytes and breast tumor tissue biopsies. The center line represents the linear regression trendline. The lines above and below the center line represent the upper and lower bounds of the 95% confidence interval around the trendline. r, correlation coefficient evaluated by the Pearson test; *p, p*-value.

Pyrosequencing was used to assess the methylation levels of *ZNF577*. The primer sequences used in this analysis were designed using Qiagen's PyroMark Assay Design 2.0 software to hybridize to CpG-free sites to ensure methylation-independent amplification. Briefly, polymerase chain reaction (PCR) was performed using standard conditions with biotinylated primers, and the Swift MaxPro thermalcycler (Esco Healthcare, Singapore) was used to prepare single-stranded PCR products according to the manufacturer's instructions. Pyrosequencing reactions and methylation quantification were performed in a PyroMark Q24 System version 2.0.7 (Qiagen), using appropriate reagents and recommended protocols. The primer sequences used were (all given 5′ > 3′): *ZNF577* prePCR forwad: GGGTAGAGGYGAGTGTTTAGAGAT, *ZNF577* pre-PCR reverse: [Btn] CTCCCTACCCCTAAAACAT; *ZNF577* seq: TTTAGTAGTGGAGATAGG). These primers allowed to quantify the methylation levels of two CpG corresponding to the target IDs cg03562414 and cg10635122 of the Infinium Human Methylation 450 BeadChip array (mapinfo 52391078 and 52391090, respectively, according to GRCh37/hg19 from UCSC Genome Browser). These CpG sites were previously identified as differentially methylated in breast tumor tissues depending on menopausal and adiposity state (10). Both CpG sites are located at the promoter region and island of *ZNF577* (Figure 1A). Promoter region was defined as the sequence from 1,500 bps upstream of transcription start site (TSS) to 1st exon (42, 43).

### Gene Expression Assessment

For data analysis, gene expression levels were normalized *GAPDH* as internal control, and they were expressed as the average value for the control group according to the 2^−ΔΔCt^ method. RT-qPCR experiments were performed in compliance with the Minimum Information for Publication of Quantitative Real-Time PCR Experiments guidelines (http://www.rdml.org/miqe).

RNA was isolated from peripheral blood mononuclear cells (PBMCs) using Trizol (Invitrogen) according to the manufacturer's recommendations. Extracted total RNA was purified with DNase treatment using a DNA-free kit as a template (Ambion) to generate first-strand cDNA synthesis using the High-Capacity cDNA Reverse Transcription Kit (Applied Biosystems). The expression of *ZNF577* was assessed using TaqMan real-time PCR and a Step One Plus system (Applied Biosystems, USA) with specific primers and probes for the *ZNF577* gene that were obtained from inventoried TaqMan Gene Expression Assays (Applied Biosystems, USA). All reactions were performed using the following cycling parameters: 50°C for 2 min, 95°C for 10 min, followed by 40 cycles of 95°C for 15 s, 60°C for 1 min. All experiments were performed in duplicate and gene expression levels were normalized using *GAPDH* as an internal control. The fold change in gene expression was calculated using the 2^−ΔΔCt^ relative quantitation method according to the manufacturer's guidelines (Applied Biosystems), and data are reported as mean ± standard error of the mean (SEM). RT-qPCR experiments were performed in compliance with the MIQE (Minimum Information for Publication of Quantitative Real-Time PCR Experiments) guidelines (http://www.rdml.org/miqe). The commercially available and prevalidated TaqMan primer/probe sets used were as follows: glyceraldehyde-3-phosphate dehydrogenase (*GAPDH*, Hs02758991_g1, Applied Biosystems) and zinc finger protein *ZNF577* (*ZNF577*, Hs00971281_m1; Applied Biosystems).

### Statistical Analyses

The sample size of the current study was calculated to obtain differences in the methylation levels of *ZNF577*. It was calculated for an α = 0.05, and a power (1-β) of 80%. The normal distribution of variables was explored using the Kolmogorov-Smirnov and the Shapiro-Wilk tests. The Chi-square (X^2^) test was used to compare the prevalence of obesity in different food consumption groups. The Student's *t*-test was used to study the differences between the groups and the differences were further evaluated by a univariant ANCOVA, adjusted for BMI and age.

The analysis of the difference in methylation levels of *ZNF577* between the different groups was performed with the two CpG sites separately and also with the mean value of the two CpG sites.

The potential association between the methylation levels of *ZNF577* with anthropometric- and body composition-parameters, and food consumption was evaluated using the Spearman coefficient test. Also, the Spearman coefficient test was performed to evaluate the correlation between *ZNF577* methylation levels in leukocytes and in breast tumor tissue biopsy. Multivariate linear regression models were fitted to assess the potential association between the methylation levels of *ZNF577* and food consumption, adjusted for BMI and age. Three regression models were performed. Model 1 included all variables of food groups consumption, model 2 included variables of vegetables, legumes and fish consumption, and model 3 included the variable of fish consumption. Additionally, a receiver operating characteristic (ROC) curve analysis was performed to prove that the regression model including fish consumption alone is the best predictive tool to identify patients with high level of *ZNF577* methylation. These results are often interpreted as negligible efficiency (< 20%), minimal (20–40%), moderate (41–60%), good (61–80%) and excellent (>80%).

Statistical analyses were performed using the SPSS version 22.0 software (SPSS Inc., Chicago, IL, USA) for Windows XP (Microsoft, Redmond, WA, USA). A *p* ≤ 0.05 was considered statistically significant.

## Results

### Characteristics of Patients

Among the 90 patients with primary breast cancer included in this study, 64 patients (71.1%) were classified as overweight/obese and 26 (28.9%) were classified to have normal-weight and 62 (68.9%) patients were postmenopausal (Table 1). The mean BMI was 25.0 ± 3.4 in premenopausal patients and 30.0 ± 5.5 in postmenopausal patients. The mean waist perimeter was 83.7 ± 12.2 for premenopausal patients and 93.8 ± 14.2 for postmenopausal patients. Likewise, the mean WHR was 0.81 ± 0.14 for premenopausal patients and 0.92 ± 0.07 for postmenopausal patients. These differences according to the menopausal state were statistically significant (*p* < 0.05). In fact, the highest BMI and waist-to-height ratio (WHtR) was observed among overweight/obese postmenopausal women (Table 1). The characteristics of tumors were not found to be different depending on the adiposity (Table 2).

**Table 1:** Anthropometric and body composition according to adiposity and menopausal state.

	**Normal-weight**			**Overweight/Obese**		
	**Premenopausal (*n* = 13)**	**Postmenopausal (*n* = 13)**	***p*-value**	**Premenopausal (*n* = 15)**	**Postmenopausal (*n* = 49)**	***p-*value**
Body weight (kg)	58.0 ± 7.2	58.6 ± 7.0	0.827	73.5 ± 8.1	77.5 ± 12.6	0.253
Height (m)	1.62 ± 0.08	1.58 ± 0.06	0.171	1.63 ± 0.07	1.56 ± 0.05	**<0.001**
BMI (kg/m^2^)	22.0 ± 1.9	23.4 ± 1.7	0.073	27.5 ± 1.9	31.7 ± 4.7	**0.001**
Age (years)	42.4 ± 4.8	59.9 ± 6.6	**<0.001**	43.5 ± 4.5	61.1 ± 8.5	**<0.001**
Waist circumference (cm)	75.3 ± 8.1	80.4 ± 9.4	0.175	90.8 ± 10.5	98.3 ± 12.7	0.061
Hip circumference (cm)	108.0 ± 14.5	99.2 ± 9.7	0.089	102.9 ± 10.7	103.6 ± 12.1	0.830
WHR	0.72 ± 0.11	0.81 ± 0.12	0.085	0.88 ± 0.13	0.96 ± 0.19	0.179
WHtR	0.46 ± 0.05	0.51 ± 0.04	**0.027**	0.55 ± 0.06	0.63 ± 0.08	**0.002**

*Data show as the mean ± standard deviation. P-value is calculated by Student's t-test. BMI, body mass index; WHR, waist-hip ratio; WHtR, waist-height ratio. Bold means statistially significant results (p < 0.05)*.

**Table 2:** Tumor characteristics and associated *p*-value chi-square test according to body mass index (BMI).

	**BMI**		
	**<25**	**≥25**	***p* chi-square**
Stage at diagnosis			0.636
I/II	15 (57.7)	43 (67.2)	
III/IV	5 (19.2)	8 (12.5)	
Unknown	6 (23.1)	13 (20.3)	
Receptor status			
Estrogen receptor (ER)			0.595
Positive	21 (80.8)	48 (75.0)	
Negative	2 (7.7)	10 (15.6)	
Unknown	3 (11.5)	6 (9.4)	
Progesterone receptor (PR)			0.604
Positive	19 (73.1)	42 (65.6)	
Negative	4 (15.4)	16 (25.0)	
Unknown	3 (11.5)	6 (9.4)	
Hercept test			0.893
Positive	2 (7.7)	7 (10.9)	
Negative	20 (76.9)	48 (75.0)	
Unknown	4 (15.4)	9 (14.1)	
Histology			0.966
Invasive ductal carcinoma	20 (76.9)	48 (75.0)	
Other	4 (15.4)	10 (15.6)	
Unknown	2 (7.7)	6 (9.4)	
Tumor size (cm)			0.343
≤ 2	13 (50.0)	41 (64.1)	
>2	11 (42.3)	17 (26.6)	
Unknown	2 (7.7)	6 (9.4)	

*Data show as the sample size (percentage) of each group according to adiposity*.

To evaluate the association between obesity-related features among breast cancer patients and dietary habits, the food frequency questionnaire was analyzed in the context of adherence to the Mediterranean diet and the consumption of the most important food groups (vegetables, legumes, fish and read meat and sausages) (Table 3). Statistical analysis post BMI adjustment yielded no significant differences in the context of adherence to the Mediterranean diet, and consumption of vegetables and legumes (Table 3). Significant differences were observed in the height, age, and waist circumference in relation to the consumption of red meat and sausages. Age of the patient and its association with fish consumption was also found to be statistically significant. Women with breast cancer who consumed higher than the recommended amounts of red meat and sausages were taller than patients who consumed lower than the recommended amounts of meat. Also, women with breast cancer who consumed higher than recommended amounts of fish were older than patients who consumed lower than the recommended amounts of fish.

**Table 3:** Association between obesity-related features among breast cancer patients and dietary habits.

	**Adherence Mediterranean diet**		**Vegetables consumption**		**Legumes consumption**		**Fish consumption**		**Read meat and sausages consumption**	
	**≤7 points (*n* = 49)**	**>7 points (*n* = 18)**	**< recommended (*n* = 50)**	**≥recommended (*n* = 19)**	**< recommended (*n* = 41)**	**≥recommended (*n* = 28)**	**< recommended (*n* = 51)**	**≥recommended (*n* = 18**	**< recommended (*n* = 35)**	**≥recommended (*n* = 33)**
Prevalence (%)	73.1	29.9	72.5	27.5	59.4	40.6	73.9	26.1	51.2	48.5
Body weight (kg)	72.3 ± 14.1	68.9 ± 8.9	71.0 ± 13.8	71.6 ± 10.4	70.8 ± 13.9	71.7 ± 11.4	70.5 ± 13.6	73.5 ± 10.2	69.1 ± 11.4	73.4 ± 14.5
Height (m)	1.60 ± 0.07	1.47 ± 0.05	1.59 ± 0.07	1.58 ± 0.07	1.59 ± 0.07	1.58 ± 0.06	1.59 ± 0.07	1.58 ± 0.04	1.57 ± 0.06	1.61 ± 0.07^*^
BMI (kg/m^2^)	28.3 ± 5.7	27.8 ± 3.2	28.1 ± 5.6	28.6 ± 3.6	28.0 ± 5.5	28.6 ± 4.5	27.9 ± 5.5	29.4 ± 3.6	28.0 ± 4.7	28.3 ± 5.8
Age (years)	53.7 ± 11.4	57.9 ± 7.3	55.0 ± 11.4	56.5 ± 9.4	54.6 ± 11.3	56.6 ± 10.3	53.6 ± 11.1	61.3 ± 7.7^*^	58.7 ± 10.5	51.3 ± 10.0^*^
Waist circumference (cm)	91.7 ± 15.3	87.8 ± 9.1	91.1 ± 14.5	89.4 ± 12.6	90.8 ± 14.3	90.1 ± 13.4	90.8 ± 15.3	89.9 ± 9.2	88.6 ± 12.7	92.1 ± 14.9^**‡**^
Hip circumference (cm)	104.7 ± 11.7	106.3 ± 11.7	106.1 ± 12.2	100.8 ± 10.2	105.0 ± 12.3	103.8 ± 11.1	105.0 ± 11.6	102.9 ± 12.6	102.5 ± 11.7	106.5 ± 11.8
WHR	0.87 ± 0.18	0.82 ± 0.14	0.84 ± 0.15	0.89 ± 0.19	0.85 ± 0.13	0.87 ± 0.21	0.86 ± 0.17	0.87 ± 0.14	0.86 ± 0.15	0.86 ± 0.18
WHtR	0.57 ± 0.10	0.56 ± 0.06	0.57 ± 0.10	0.57 ± 0.08	0.57 ± 0.10	0.57 ± 0.09	0.57 ± 0.10	0.57 ± 0.06	0.56 ± 0.09	0.57 ± 0.10
Prevalence of obesity^†^	68.9% (31)	80.0% (12)	67.4% (31)	81.3% (13)	65.8% (25)	79.2% (19)	66.7% (32)	85.5% (12)	69.7% (23)	71.4% (20)

*Data show the mean ± standard deviation*.

† *Data show the percentage (sample size)*.

* *Statistically significant (p < 0.05) differences respect to < recommended evaluated by Student's t-test within the consumption of each food group*.

‡ *Statistically significant (p < 0.05) differences were evaluated with univariant ANCOVA adjusted for body mass index (BMI) and age. WHR, waist to hip ratio; WHtR, waist-to-height ratio*.

### Methylation and Expression Levels of *ZNF577* Based on Adiposity, Menopausal Status, and Food Consumption

**Figure 2:**
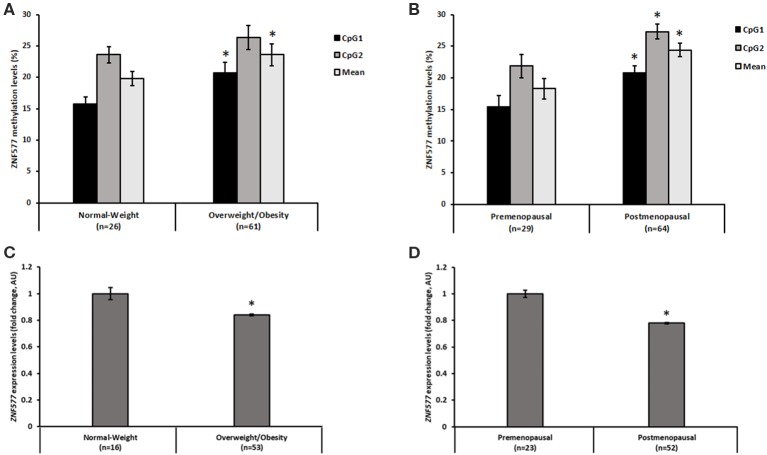
Methylation and gene expression of *ZNF577* according to adiposity and menopausal state. Methylation levels of *ZNF577* in leukocytes from patients in this study according to **(A)** adiposity, and **(B)** menopausal state. Gene expression of *ZNF577* in peripheral blood mononuclear cells (PBMCs) according to **(C)** adiposity, and **(D)** menopausal state. Data are presented as the mean; error bars represent the standard error. Asterisk (*) denotes statistically significant differences (*p* < 0.05) in relation to normal-weight or premenopausal state evaluated by Student's *t*-test.

A direct correlation was found between *ZNF577* methylation levels of paired leukocytes and breast tumor tissue biopsies from 8 breast cancer women considering data from CpG1, CpG2, and mean (Figure 1B; *r* = 0.62; *p* = 0.001). Accordingly, *ZNF577* methylation levels in the leukocytes from women with breast cancer were higher in patients with obesity than in patients with normal-weight (*p* = 0.002; Figure 2A), and in postmenopausal than in premenopausal women (*p* = 0.022; Figure 2B). The results were in a similar direction of that previously published in breast tumor tissue biopsy (10).

In order to evaluate the effect of promoter methylation on its function, the expression levels of *ZNF577* was assessed and an inverse correlation was found with statistical significance between transcript levels and methylation levels in CpG1 and mean of both CpGs (CpG1: *r* = −0.28; *p* = 0.023, CpG2: *r* = −0.18; *p* = 0.143, Mean: *r* = −024; *p* = 0.05). In fact, the expression of this gene in the leukocytes from women with breast cancer was lower in women with obesity than that in the women with normal-weight (*p* = 0.039; Figure 2C), and lower in postmenopausal women than that in the premenopausal women (*p* = 0.007; Figure 2D).

**Figure 3:**
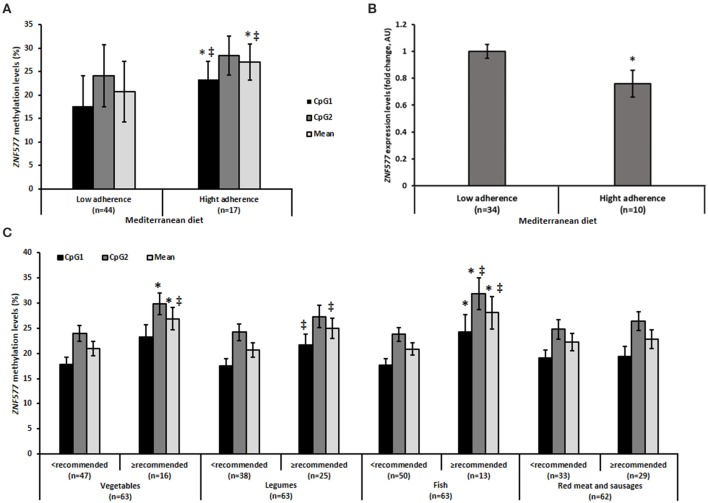
Methylation and gene expression of *ZNF577* according to dietary patterns. **(A)** Mehylation levels of *ZNF577* in leukocytes from patients in this study according to adherence to the Mediterranean diet. **(B)** Gene expression of *ZNF577* in peripheral blood mononuclear cells (PBMCs) in patients in this study according to adherence to the Mediterranean diet. **(C)** Methylation levels of *ZNF577* in leukocytes from patients in this study according to food groups of the Mediterranean diet. Data are presented as the mean; error bars represent the standard error. Asterisk (*) denotes statistically significant differences (*p* < 0.05) in relation to low adherences to the Mediterranean diet or lower consumption than recommended of Mediterranean diet food groups evaluated by Student's *t*-test. ^‡^denotes statistically significant differences (*p* < 0.05) in relation to low adherence to the Mediterranean diet or lower consumption than recommended of Mediterranean diet food groups evaluated by univariant ANCOVA adjusted for body mass index (BMI) and age.

Upon analysis of the methylation levels of *ZNF577* in relation to adherence to the Mediterranean diet, it was observed that women who have greater adherence to the diet showed higher levels of methylation, as evidenced by the statistically significant differences in CpG1 and in the mean of the two evaluated CpG sites (Figure 3A). Notably the differences in methylation levels according to the adherence to the Mediterranean diet was inversely correlated with differences in the gene expression of *ZNF577*. These differences were observed with statistical significance (*p* = 0.007, Figure 3B).

Further analysis was performed by individual evaluation of the food groups of the Mediterranean diet in relation to the methylation levels of *ZNF577*. Statistically significant differences were observed in specific items (Figure 3C). The methylation levels of *ZNF577* were higher in women who consumed more than the recommended amounts of vegetables and fish. The results obtained were statistically significant in CpG2 and in the mean of the two evaluated CpGs in vegetable consumption, in CpG1 and in the mean of CpGs adjusted for age and BMI in the consumption of legumes, and in CpG1, CpG2 and in the mean of CpGs in fish consumption. No statistically significant results were obtained in relation to the consumption of red meat and sausages (Figure 3C). Despite the differences in methylation levels according to specific foods, differences in the expression of *ZNF577* depending on specific foods were not detected with statistical significance (Supplementary Table 1).

**Figure 4:**
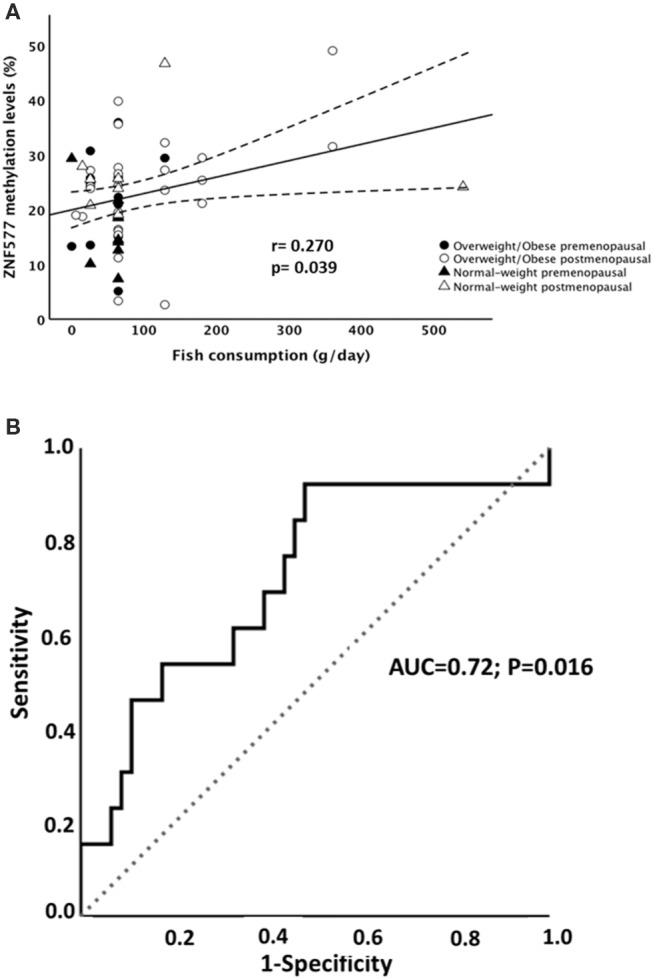
Association between methylation levels of *ZNF577* and fish consumption. **(A)** Scatterplot representing the association between *ZNF577* methylation levels in leukocytes from patients in this study and fish consumption in grams per day. The center line represents the linear regression trendline. The lines above and below the center line represent the upper and lower bounds of the 95% confidence interval around the trendline. r, correlation coefficient evaluated by the Pearson test; *p, p*-value. **(B)** Receiver operating characteristic (ROC) curves for *ZNF577* methylation levels to discriminate the patients according to the fish consumption.

Particularly, methylation levels of *ZNF577* correlated directly with fish consumption (Figure 4A), while no statistically significant correlation was found between methylation levels and consumption of vegetables, legumes, and meat. Moreover, linear regression models revealed that 16% of the variability in the *ZNF577* methylation levels, adjusted for age and BMI, was conjointly explained by the consumption of vegetables, legumes, and fish (Table 4). In fact, a receiver operating characteristic (ROC) curve determine the ability of *ZNF577* methylation levels in leukocytes to discriminate patients according to the consumption of fish with an area under the ROC curve (AUC) of 0.72 (*p* < 0.001) (Figure 4B).

**Table 4:** Independent effects of vegetables, legumes, fish and read met consumption on Methylation levels of ZNF577 in leukocytes from breast cancer women at the moment of diagnosis.

	**Standardized coefficients β (95% CI)**	***P-value***
**Model 1**		
Vegetables consumption	0.159 (−2.4;9.21)	0.246
Legumes consumption	0.201 (−1.25;8.88)	0.137
Fish consumption	0.183 (−2.21;10.43)	0.197
Read meat and sausage consumption	0.148 (−2.38;7.95)	0.284
*Corrected R^2^ = 0.15*		0.027
**Model 2**		
Vegetables consumption	0.168 (−2.18;9.33)	0.218
Legumes consumption	0.187 (−1.44;8.50)	0.160
Fish consumption	0.189 (−1.99;10.48)	0.178
*Corrected R ^2^= 0.16*		0.019
**Model 3**		
Fish consumption	0.278 (0.18;12.34)	0.044
*Corrected R^2^=* −*0.11*		0.029

*Adjusted for age and body mass index (BMI)*.

## Discussion

Obesity-related breast cancer has been previously associated with the methylation levels of the promoter region of *ZNF577*, a specific methylation pattern identified among a potential epi-signature of breast cancer in obese women (10). In the current study, the methylation levels of *ZNF577* were measured in the blood leukocytes, to evaluate if the identified methylation levels in breast tumors can be reflected in a non-invasive and easily obtained source of DNA. In this context, it was demonstrated that the methylation levels of *ZNF577* were also observed in association with adiposity and menopausal status, and the direction of differences in methylation levels were inverse to that in expression of this gene in the blood leukocytes. Dietary habits are also a risk factor for developing cancer and obesity, as well as both factors were related to the regulation of the methylation profile (44–46). Thus, when the methylation levels of *ZNF577* were evaluated according to the dietary habits, breast cancer patients who adhered to a Mediterranean diet and who specifically consumed higher amounts of vegetables, legumes, and fish showed the highest levels of methylation in *ZNF577*, independently of menopausal and obesity status. As far as we know, the current work is the first to evaluate the effect of a food consumption pattern on the methylation level of the ZNF genes in women with breast cancer. Therefore, further studies will be needed to elucidate if the effect of dietary factors on the modulation of methylation levels of *ZNF577* is also reflected in the function of this gene, and the role of this regulation on breast cancer progression.

Adherence to Mediterranean diet has been associated with a lower incidence of cancer (47–49). In fact, specific compounds contained in foods included in the Mediterranean diet are able to modulate the methylation levels of several genes in the context of disorders like cardiovascular disease (50–52), stroke (51, 53), and cancer (54, 55). In agreement with these previous reports, the adherence to Mediterranean diet has been related to higher methylation levels of *ZNF577*. When the association between methylation levels of *ZNF577* were evaluated according to the consumption of specific foods included in the Mediterranean diet, the highest methylation levels of *ZNF577* were shown in the breast cancer patients who consumed the recommended amounts of vegetables, legumes, and fish. However, differences were not observed in the *ZNF577* methylation profile in association with meat consumption.

The International Agency for Research on Cancer (WHO-IARC) classified consumption of red meat as “probably carcinogenic to humans” (56). This classification is based mainly on the evidence found with colon cancer; however, previous studies have observed that a high consumption of red meat has been also associated with an increased risk of breast cancer and other types of cancer (57, 58).

Relevantly, the association analysis demonstrated that the consumption of fish was the highest contributor to the modulation of the *ZNF577* methylation levels. These results are in agreement with the beneficial effects of fish consumption on the promotion of health. Fish contain bioactive compounds such as omega-3 polyunsaturated fatty acids (n-3 FAs), specially eicosapentaenoic acid (EPA) and docosahexaenoic acid (DHA). These compounds have significant multiple antitumor activities and have been used as immune-nutrients (59, 60). Epigenetic modifications are among the mechanisms by which the fish-related bioactive compounds induce healthy effects. Differentially methylated CpG sites have been identified in the blood leukocytes from overweight and obese subjects after a 6-week supplementation with n-3 FAs (61) and also after 6 months of n-3 FAs supplementation in patients with Alzheimer's disease (62).

Because Mediterranean diet and fish consumption is a protective factor and obesity is a risk factor of breast cancer, the association between high *ZNF577* methylation levels and Mediterranean diet adherence or fish consumption together with obesity and menopausal state found in the current study could be considered counterintuitive and requires further investigation.

The strength and novelty of the current work has been represented by the use of blood cells, since circulating leukocytes constitute an easy and non-invasive tool to obtain nucleic acids in a clinical setting and perform nutrigenomic/nutriepigenomic studies, instead of invasive biopsies of the target tissue. In fact, previous results observed in the biopsies from breast cancer tumors, in association with obesity and menopausal state (10), were similar to the results obtained in leukocytes in the present study. The magnitude of the DNA methylation differences between the groups may be considered small. An explanation may be attributed to the cellular heterogeneity of the sample used for evaluating the methylation levels of a gene in a single cell type. Also, it may be because patients with the same pathology were grouped only according to food consumption patterns based on established recommendations, instead of evaluating the effect of a specific intervention in a longitudinal study. On the other hand, clinical and pathological characteristics of tumors and therapeutic strategies were not used to stratify and explore the population study and these parameters could influence the methylation levels. However, the clinical characteristics of tumors in the studied cohort were mostly homogeneous with most of tumors were invasive ductal carcinoma in first state, small size, positive RE and PR and negative hercept test. Therefore, the results of the present study are of the foremost relevance because differences in the methylation levels were observed under a narrow range of intergroup differences in the nutritional behavior.

In conclusion, the current work demonstrates that the methylation pattern of *ZNF577* previously identified in breast cancer tissue according to the adiposity and menopausal status (10), can be also detected in leukocytes from the peripheral blood. Relevantly, a specific dietary habit such as adherence to Mediterranean diet specifically fish consumption appears to modulate the methylation levels of *ZNF577* in blood leukocytes independently of the BMI and age. Therefore, *ZNF577* may be a biomarker for the effect of environmental factors such as adiposity, age, and diet on breast cancer, and a suitable therapeutic target in precision nutrition and medicine.

## Data Availability Statement

The datasets generated for this study are available on request to the corresponding author.

## Ethics Statement

The studies involving human participants were reviewed and approved by Comité Ético de Investigación Clínica, CEIC, de Galicia; Ref:2009/076. The patients/participants provided their written informed consent to participate in this study.

## Author Contributions

Conceptualization: PL, FC, and AC. Patients recruitment and sample collection: JC and RL-L. Bioinformatics analysis of the methylation data: AD-L, JS, and MM-G. Gene expression experiments and analysis: AI and MC. Data curation: PL. Formal analysis: PL. Investigation: PL, FC, and AC. Methodology and supervision: AC. Writing—original draft: PL and AC. Writing—review and editing: PL, AI, AD-L, MC, MM-G, JS, JC, RL-L, FC, and AC.

## Conflict of Interest

The authors declare that the research was conducted in the absence of any commercial or financial relationships that could be construed as a potential conflict of interest. The handling Editor declared a past co-authorship with the author MM-G.

## References

[1] Sun YS Zhao Z Yang ZN Xu F Lu HJ Zhu ZY. Risk factors and preventions of breast cancer. Int J Biol Sci. (2017) 13:1387–97. 10.7150/ijbs.21635 29209143 PMC5715522

[2] Garcia-Estevez L Moreno-Bueno G. Updating the role of obesity and cholesterol in breast cancer. Breast Cancer Res. (2019) 21:35. 10.1186/s13058-019-1124-1 30823902 PMC6397485

[3] Lorincz AM Sukumar S. Molecular links between obesity and breast cancer. Endocr Relat Cancer. (2006) 13:279–92. 10.1677/erc.1.00729 16728564

[4] van Gemert WA Lanting CI Goldbohm RA van den Brandt PA Grooters HG Kampman E. The proportion of postmenopausal breast cancer cases in the Netherlands attributable to lifestyle-related risk factors. Breast Cancer Res Treat. (2015) 152:155–62. 10.1007/s10549-015-3447-7 26044369 PMC4469298

[5] Crujeiras AB Cueva J Vieito M Curiel T López-López R Pollán M. Association of breast cancer and obesity in a homogeneous population from Spain. J Endocrinol Invest. (2012) 35:681–5. 10.3275/8370 22522745

[6] Bultman SJ. Interplay between diet, gut microbiota, epigenetic events, and colorectal cancer. Mol Nutr Food Res. (2017) 61:10. 10.1002/mnfr.201500902 27138454 PMC5161716

[7] Kubota T. Epigenetic effect of environmental factors on neurodevelopmenal disorders. Nihon Eiseigaku Zasshi. (2016) 71:200–7. 10.1265/jjh.71.200 27725423

[8] Baxter E Windloch K Gannon F Lee JS. Epigenetic regulation in cancer progression. Cell Biosci. (2014) 4:45. 10.1186/2045-3701-4-45 25949794 PMC4422217

[9] Crujeiras AB Morcillo S Diaz-Lagares A Sandoval J Castellano-Castillo D Torres E. Identification of an episignature of human colorectal cancer associated with obesity by genome-wide DNA methylation analysis. Int J Obes. (2019) 43:176–88. 10.1038/s41366-018-0065-6 29717273

[10] Crujeiras AB Diaz-Lagares A Stefansson OA Macias-Gonzalez M Sandoval J Cueva J. Obesity and menopause modify the epigenomic profile of breast cancer. Endocr Relat Cancer. (2017) 24:351–63. 10.1530/ERC-16-0565 28442560

[11] Crujeiras AB Diaz-Lagares A Sandoval J Milagro FI Navas-Carretero S Carreira MC. DNA methylation map in circulating leukocytes mirrors subcutaneous adipose tissue methylation pattern: a genome-wide analysis from non-obese and obese patients. Sci Rep. (2017) 7:41903. 10.1038/srep41903 28211912 PMC5314866

[12] Crujeiras AB Diaz-Lagares A Moreno-Navarrete JM Sandoval J Hervas D Gomez A. Genome-wide DNA methylation pattern in visceral adipose tissue differentiates insulin-resistant from insulin-sensitive obese subjects. Transl Res. (2016) 178:13–24. 10.1016/j.trsl.2016.07.002 27477082

[13] Severson PL Tokar EJ Vrba L Waalkes MP Futscher BW. Coordinate H3K9 and DNA methylation silencing of ZNFs in toxicant-induced malignant transformation. Epigenetics. (2013) 8:1080–88. 10.4161/epi.25926 23974009 PMC3891689

[14] Xiang H Zhong Z-X Peng Y-D Jiang S-W. The emerging role of Zfp217 in Adipogenesis. Int J Mol Sci. (2017) 18:1367. 10.3390/ijms18071367 28653987 PMC5535860

[15] Kang S Akerblad P Kiviranta R Gupta RK Kajimura S Griffin MJ. Regulation of early adipose commitment by Zfp521. PLoS Biol. (2012) 10:e1001433. 10.1371/journal.pbio.1001433 23209378 PMC3507953

[16] Gupta RK Arany Z Seale P Mepani RJ Ye L Conroe HM. Transcriptional control of preadipocyte determination by Zfp423. Nature. (2010) 464:619–23. 10.1038/nature08816 20200519 PMC2845731

[17] Jia Y Yuan L Hu W Luo Y Suo L Yang M. Zinc-finger BED domain-containing 3 (Zbed3) is a novel secreted protein associated with insulin resistance in humans. J Intern Med. (2014) 275:522–33. 10.1111/joim.12170 24283382

[18] Scherneck S Vogel H Nestler M Kluge R Schürmann A Joost HG. Role of zinc finger transcription factor Zfp69 in body fat storage and diabetes susceptibility of mice. Results Probl Cell Differ. (2010) 52:57–68. 10.1007/978-3-642-14426-4_6 20865372

[19] Liu G Zhou L Zhang H Chen R Zhang Y Li L. Regulation of hepatic lipogenesis by the zinc finger protein Zbtb20. Nat Commun. (2017) 8:14824. 10.1038/ncomms14824 28327662 PMC5364431

[20] Fernandes GW Bocco BMLC Fonseca TL McAninch EA Jo S Lartey LJ. The Foxo1-inducible transcriptional repressor Zfp125 causes hepatic steatosis and hypercholesterolemia. Cell Rep. (2018) 22:523–34. 10.1016/j.celrep.2017.12.053 29320745 PMC6474669

[21] Fujita R Yoshioka K Seko D Suematsu T Mitsuhashi S Senoo N. Zmynd17 controls muscle mitochondrial quality and whole-body metabolism. FASEB J. (2018) 32:5012–25. 10.1096/fj.201701264R 29913553

[22] Choi WI Yoon JH Song JY Jeon BN Park JM Koh DI. Zbtb7c is a critical gluconeogenic transcription factor that induces glucose-6-phosphatase and phosphoenylpyruvate carboxykinase 1 genes expression during mice fasting. Biochim Biophys Acta Gene Regul Mech. (2019) 1862:643–56. 10.1016/j.bbagrm.2019.04.001 30959128

[23] Clinkenbeard EL Turpin C Jiang J Peterson ML Spear BT. Liver size and lipid content differences between BALB/c and BALB/cJ mice on a high-fat diet are due, in part, to Zhx2. Mamm Genome. (2019) 30:226–36. 10.1007/s00335-019-09811-6 31321500 PMC6761023

[24] Caracciolo V Young J Gonzales D Ni Y Flowers SJ Summer R. Myeloid-specific deletion of Zfp36 protects against insulin resistance and fatty liver in diet-induced obese mice. Am J Physiol Endocrinol Metab. (2018) 315:E676–93. 10.1152/ajpendo.00224.2017 29509432 PMC6230714

[25] Williams EJ Baines KJ Berthon BS Wood LG. Effects of an encapsulated fruit and vegetable juice concentrate on obesity-induced systemic inflammation: a randomised controlled trial. Nutrients. (2017) 9:116. 10.3390/nu9020116 28208713 PMC5331547

[26] Blüher M. Obesity: global epidemiology and pathogenesis. Nat Rev Endocrinol. (2019) 15:288–98. 10.1038/s41574-019-0176-8 30814686

[27] Bray GA Heisel WE Afshin A Jensen MD Dietz WH Long M. The science of obesity management: an endocrine society scientific statement. Endocr Rev. (2018) 39:79–132. 10.1210/er.2017-00253 29518206 PMC5888222

[28] Dick KJ Nelson CP Tsaprouni L Sandling JK Aïssi D Wahl S. DNA methylation and body-mass index: a genome-wide analysis. Lancet. (2014) 383:1990–8. 10.1016/S0140-6736(13)62674-4 24630777

[29] Nicoletti CF Pinhel MS Noronha NY Jácome A Crujeiras AB Nonino CB. Association of MFSD3 promoter methylation level and weight regain after gastric bypass: assessment for 3 y after surgery. Nutrition. (2020) 70:110499. 10.1016/j.nut.2019.04.010 31655468

[30] Ollikainen M Ismail K Gervin K Kyllönen A Hakkarainen A Lundbom J. Genome-wide blood DNA methylation alterations at regulatory elements and heterochromatic regions in monozygotic twins discordant for obesity and liver fat. Clin Epigenetics. (2015) 7:39. 10.1186/s13148-015-0073-5 25866590 PMC4393626

[31] Chambers JC Loh M Lehne B Drong A Kriebel J Motta V. Epigenome-wide association of DNA methylation markers in peripheral blood from Indian Asians and Europeans with incident type 2 diabetes: a nested case-control study. Lancet Diabetes Endocrinol. (2015) 3:526–34. 10.1016/S2213-8587(15)00127-8 26095709 PMC4724884

[32] Zheng LD Linarelli LE Liu L Wall SS Greenawald MH Seidel RW. Insulin resistance is associated with epigenetic and genetic regulation of mitochondrial DNA in obese humans. Clin Epigenetics. (2015) 7:60. 10.1186/s13148-015-0093-1 26110043 PMC4479353

[33] Zhang FF Cardarelli R Carroll J Zhang S Fulda KG Gonzalez K. Physical activity and global genomic DNA methylation in a cancer-free population. Epigenetics. (2011) 6:293–9. 10.4161/epi.6.3.14378 21178401 PMC3092677

[34] Zhang FF Morabia A Carroll J Gonzalez K Fulda K Kaur M. Dietary patterns are associated with levels of global genomic DNA methylation in a cancer-free population. J Nutr. (2011) 141:1165–71. 10.3945/jn.110.134536 21525250 PMC3095144

[35] Zhang FF Cardarelli R Carroll J Fulda KG Kaur M Gonzalez K. Significant differences in global genomic DNA methylation by gender and race/ethnicity in peripheral blood. Epigenetics. (2011) 6:623–9. 10.4161/epi.6.5.15335 21739720 PMC3230547

[36] Crujeiras AB Campion J Díaz-Lagares A Milagro FI Goyenechea E Abete I. Association of weight regain with specific methylation levels in the NPY and POMC promoters in leukocytes of obese men: a translational study. Regul Pept. (2013) 186:1–6. 10.1016/j.regpep.2013.06.012 23831408

[37] Milagro FI Campión J Cordero P Goyenechea E Gómez-Uriz AM Abete I. A dual epigenomic approach for the search of obesity biomarkers: DNA methylation in relation to diet-induced weight loss. FASEB J. (2011) 25:1378–89. 10.1096/fj.10-170365 21209057

[38] WHO Obesity: preventing and managing the global epidemic. Report of a WHO consultarion. World Health Organ Tech Rep Ser. (2000) 894:1–253. 11234459

[39] Palma I Farran A Cantós D Tablas de composición de alimentos por medidas caseras de consumo habitual en España. Barcelona: McGRAW-HILL (2008).

[40] Dapcich V Salvador G Ribas L Pérez C Aranceta J Serra L Guía de la alimentación saludable. Senc. (2004).

[41] Schröder H Fitó M Estruch R Martínez-González MA Corella D Salas-Salvadó J. A short screener is valid for assessing mediterranean diet adherence among older Spanish men and women. J Nutr. (2011) 141:1140–45. 10.3945/jn.110.135566 21508208

[42] Sandoval J Heyn H Moran S Serra-Musach J Pujana MA Bibikova M. Validation of a DNA methylation microarray for 450,000 CpG sites in the human genome. Epigenetics. (2011) 6:692–702. 10.4161/epi.6.6.16196 21593595

[43] Diaz-Lagares A Crujeiras AB Lopez-Serra P Soler M Setien F Goyal A. Epigenetic inactivation of the p53-induced long noncoding RNA TP53 target 1 in human cancer. Proc Natl Acad Sci USA. (2016) 113:E7535–44. 10.1073/pnas.1608585113 27821766 PMC5127373

[44] Lima RPA do Nascimento RAF Luna RCP Persuhn DC da Silva AS da Conceição Rodrigues Gonçalves M Effect of a diet containing folate and hazelnut oil capsule on the methylation level of the ADRB3 gene, lipid profile and oxidative stress in overweight or obese women. Clin Epigenetics. (2017) 9:110 10.1186/s13148-017-0407-6 29046732 PMC5640916

[45] Campión J Milagro F Martínez JA Epigenetics and obesity. Prog Mol Biol Transl Sci. (2010) 291–347. 10.1016/B978-0-12-375003-7.00011-X 21036330

[46] Donovan MG Wren SN Cenker M Selmin OI Romagnolo DF Dietary fat and obesity as modulators of BC risk: focus on DNA methylation. Br J Pharmacol. (2019) 177:1331–50. 10.1111/bph.14891 PMC7056465 31691272

[47] Schwingshackl L Schwedhelm C Galbete C Hoffmann G. Adherence to mediterranean diet and risk of cancer: an updated systematic review and meta-analysis. Nutrients. (2017) 9:1063. 10.3390/nu9101063 28954418 PMC5691680

[48] Hernáez Á Estruch R. The mediterranean diet and cancer: what do human and molecular studies have to say about it? Nutrients. (2019) 11:2155. 10.3390/nu11092155 31505794 PMC6769497

[49] Turati F Carioli G Bravi F Ferraroni M Serraino D Montella M. Mediterranean diet and breast cancer risk. Nutrients. (2018) 10:326. 10.3390/nu10030326 29518016 PMC5872744

[50] Kalea AZ Drosatos K Buxton JL. Nutriepigenetics and cardiovascular disease. Curr Opin Clin Nutr Metab Care. (2018) 21:252–9. 10.1097/MCO.0000000000000477 29847446 PMC6432651

[51] Tuttolomondo A Simonetta I Daidone M Mogavero A Ortello A Pinto A. Metabolic and vascular effect of the mediterranean diet. Int J Mol Sci. (2019) 20:326. 10.3390/ijms20194716 31547615 PMC6801699

[52] Arpón A Riezu-Boj JI Milagro FI Marti A Razquin C Martínez-González MA. Adherence to Mediterranean diet is associated with methylation changes in inflammation-related genes in peripheral blood cells. J Physiol Biochem. (2016) 73:445–55. 10.1007/s13105-017-0552-6 28181167

[53] Corella D Ordovás JM. How does the Mediterranean diet promote cardiovascular health? Current progress toward molecular mechanisms: gene-diet interactions at the genomic, transcriptomic, and epigenomic levels provide novel insights into new mechanisms. Bioessays. (2014) 36:526–37. 10.1002/bies.201300180 24706458

[54] Fasanelli F Giraudo MT Vineis P Fiano V Fiorito G Grasso C. DNA methylation, colon cancer and Mediterranean diet: results from the EPIC-Italy cohort. Epigenetics. (2019) 14:977–88. 10.1080/15592294.2019.1629230 31179817 PMC6691992

[55] Barchitta M Maugeri A Quattrocchi A Barone G Mazzoleni P Catalfo A. Mediterranean diet and particulate matter exposure are associated with LINE-1 methylation: results from a cross-sectional study in women. Front Genet. (2018) 9:514. 10.3389/fgene.2018.00514 30425730 PMC6218419

[56] Bouvard V Loomis D Guyton KZ Grosse Y Ghissassi F El Benbrahim-Tallaa L. Carcinogenicity of consumption of red and processed meat. Lancet Oncol. (2015) 1599–600. 10.1016/S1470-2045(15)00444-1 26514947

[57] Diallo A Deschasaux M Latino-Martel P Hercberg S Galan P Fassier P. Red and processed meat intake and cancer risk: results from the prospective nutriNet-santé cohort study. Int J Cancer. (2018) 142:230–7. 10.1002/ijc.31046 28913916

[58] Farvid MS Stern MC Norat T Sasazuki S Vineis P Weijenberg MP. Consumption of red and processed meat and breast cancer incidence: a systematic review and meta-analysis of prospective studies. Int J Cancer. (2018) 143:2787–99. 10.1002/ijc.31848 30183083 PMC8985652

[59] Huang Q Mo M Zhong Y Yang Q Zhang J Ye X. Anticancer role of omega-3 polyunsaturated fatty acids was closely associated with the increase in genomic DNA hydroxymethylation. Anticancer Agents Med Chem. (2019) 19:330–6. 10.2174/1871520618666181018143026 30338745

[60] Eltweri AM Thomas AL Metcalfe M Calder PC Dennison AR Bowrey DJ. Potential applications of fish oils rich in omega-3 polyunsaturated fatty acids in the management of gastrointestinal cancer. Clin Nutr. (2017) 36:65–78. 10.1016/j.clnu.2016.01.007 26833289

[61] Tremblay BL Guénard F Rudkowska I Lemieux S Couture P Vohl M-C. Epigenetic changes in blood leukocytes following an omega-3 fatty acid supplementation. Clin Epigenetics. (2017) 9:43. 10.1186/s13148-017-0345-3 28450971 PMC5405524

[62] Karimi M Vedin I Freund Levi Y Basun H Faxén Irving G Eriksdotter M. DHA-rich n-3 fatty acid supplementation decreases DNA methylation in blood leukocytes: the OmegAD study. Am J Clin Nutr. (2017) 106:1157–65. 10.3945/ajcn.117.155648 28855224

