# Supplementary material for: ZNF577 Methylation Levels in Leukocytes From Women With Breast Cancer Is Modulated by Adiposity, Menopausal State, and the Mediterranean Diet
Source: Front Endocrinol (Lausanne). 2020 Apr 23;11:245. doi: 10.3389/fendo.2020.00245 (PMC7191069; doi:10.3389/fendo.2020.00245)
Supplement: Supplementary file 1 [file Table_1.DOC]

**Supplementary table 1. Association between ZNF577 expression levels leukocytes from breast cancer women and food groups consumption.**

|  | **ZNF577 expression levels (fold change, AU)** | ***p*-value** |
| --- | --- | --- |
| Vegetables |  | 0.082 |
| <recommended (n=34) | 1.00±0.06 |  |
| ≥recommended (n=11) | 0.84±0.10 |  |
| Legumes |  | 0.566 |
| <recommended (n=28) | 1.05±0.07 |  |
| ≥recommended (n=17) | 0.99±0.09 |  |
| Fish |  | 0.317 |
| <recommended (n=37) | 1.00±0.06 |  |
| ≥recommended (n=8) | 1.20±0.14 |  |
| Read meat and sausages |  | 0.822 |
| <recommended (n=22) | 1.07±0.09 |  |
| ≥recommended (n=22) | 1.10±0.09 |  |

Data show the mean±standard error. P-value is from univariant ANCOVA adjusted for body mass index (BMI) and age. Vegetables: 200 g/ration 2 times/day; legumes: 80 g/ration in raw 2 times/week; fish: 150 g/ration 3 times/week; read meat and sausages: 125 g/ration 3 times/week (38).
